# Sacroiliac joint fusion VS conservative management for chronic low back pain attributed to the sacroiliac joint: A protocol for systematic review and meta analysis

**DOI:** 10.1097/MD.0000000000023223

**Published:** 2020-11-13

**Authors:** Li-Ye Chen, Hao-Dong Liang, Qi-Ning Qin, Tian-Zhao Tian, Bao-Xin Liu, Min Shi, Ying-Feng Cai

**Affiliations:** Department of Orthopaedics, The Affiliated TCM Hospital of Guangzhou Medical University, Guangzhou 510130, People's Republic of China.

**Keywords:** low back pain, protocol, sacroiliac joint fusion, systematic review

## Abstract

**Introduction:**

Low back pain (LBP) is high prevalent and it is the leading cause of years lived with disability in both developed and developing countries. The sacroiliac joint (SIJ) is a common reason that caused LBP. At present, the treatment of chronic LBP attributed to SIJ is mainly conservative treatment and surgical treatment. However, there are still controversies between the 2 treating methods, and there is no recognized standard of treatment or surgical indications. Recent publications indicated that minimally invasive sacroiliac joint arthrodesis was safe and more effective improving pain, disability, and quality of life compared with conservative management in 2 years follow-up, which re-raise the focus of sacroiliac joints fusion. This paper will systematically review the available evidence, comparing the effectiveness of sacroiliac joint fusion and conservative therapy for the treatment of gait retraining for patients suffered from LBP attributed to the sacroiliac joint.

**Method and analysis:**

A systematic review and meta-analysis of relevant studies in Pubmed, Embase, SCOPUS, and Cochrane Library will be synthesized. Inclusion criteria will be studies evaluating clinical outcomes (i.e., changes to pain and/or function) comparing sacroiliac joint fusion and conservative therapy in populations sacroiliac join related LBP; studies with less than 10 participants in total will be excluded. The primary outcomes measured will be pain score, Oswestry Disability Index (ODI), and adverse events during treatment. Review Manager (Revman; Version 5.3) software will be used for data synthesis, sensitivity analysis, meta-regression, subgroup analysis, and risk of bias assessment. A funnel plot will be developed to evaluate reporting bias and Begg and Egger tests will be used to assess funnel plot symmetries. We will use the Grading of Recommendations Assessment, Development and Evaluation system to assess the quality of evidence.

**Ethics and dissemination:**

Our aim is to publish this systematic review and meta-analysis in a peer-reviewed journal. Our findings will provide information comparing the efficacy and safety comparing sacroiliac joint fusion and non-surgical treatment for patients with LBP attributed to the sacroiliac joint. This review will not require ethical approval as there are no issues about participant privacy.

**Strength and limitations**

- It is a review that included most recent studies that compared sacroiliac joint fusion and non-surgical treatment for patients with LBP attributed to the sacroiliac joint.
- The Cochrane Collaboration tool and The Grading of Recommendations Assessment, Development and Evaluation will be used to further evaluate study findings.
- Methodological and clinical heterogeneities will be exit based on the varied treatment including different surgical methods (open or minimal invasive) and conservative treatments (intra-articular steroid injections and physical therapy) in both groups of studies. Moreover, varied functional assessing methods (step rate, foot strike, treadmill or ground, training session, time) in included studies can contribute to heterogeneities.
- Different surgical approaches and duration of conservative treatment may not be comparable.

## 1 Introduction

Low back pain (LBP) is a common disease that can affect the patient's social life and work and even lead to disability.^[1]^ Thought to be a frequent source of LBP, the sacroiliac joint increasingly raised doctors’ attention and it is thought to be involved in 15% to 30% of all patients with chronic low back pain.^[2–5]^ The burden of disease associated with sacroiliac joint (SIJ) pain is at least as high as that associated with other musculoskeletal conditions such as hip osteoarthritis, degenerative spondylolisthesis, or spinal stenosis, conditions that are often treated surgically.^[6,7]^ The treatment of this disease includes conservative and surgical treatment. Conservative treatments are often symptomatic treatment, such as wearing waist circumference, articular steroid injection, acupuncture, massage, etc, but the literature reports that the effective rate of these treatments is low, only about 50% of patients’ symptoms were relieved.^[8]^ The surgical treatment of this disease has been proposed since 1900, which was open sacroiliac joint fusion. Through continuous development of surgical technique and surgical instruments, triangular tantalum screw internal fixation of the sacroiliac joint has become the most common surgical method.^[8]^ Related studies have reported that it can achieve good clinical efficacy.^[9–12]^ However, the best treatment for such diseases is still controversial. Previous systematic reviews have described sacroiliac joint fusion, which has significant clinical effects in relieving pain and improving symptoms, with fewer complications in mid to long-term follow-up.^[13]^ According to the relevant systematic reviews, there is no statistical difference in the effect of conservative treatment compared with surgical treatment, and the former showed a lower incidence of complications.^[14]^ Recent published literature points out the opposite view that surgical treatment can relieve pain and improve function better than conservative treatment.^[15–17]^ Therefore, a systematic review of recently published clinical evidence is necessary. In our study, we planned to conduct a systematic review and a meta-analysis to evaluate the evidence from all available randomized controlled trials (RCTs) that evaluate effect of sacroiliac joint fusion for relieving pain and improving function of low back.

## 2 Method

**Figure 1:**
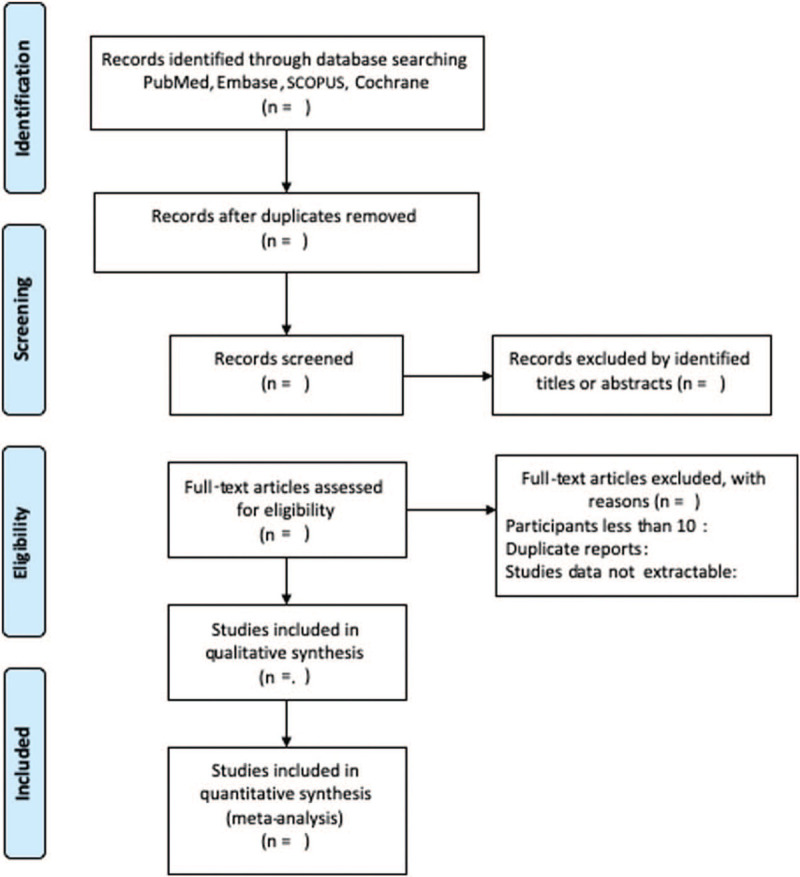
Flow diagram of the relevant study selection process.

We will conduct a systematic review and if available, meta-analysis will be performed to identify relevant studies involving sacroiliac joint fusion and LBP in electronic databases (Fig. 1). Two reviewers independently searched the electronic databases including Pubmed, Embase, SCOPUS, and Cochrane Library up to December 2020 using the following keywords and their combinations: “sacroiliac fusion,” “minimally invasive sacroiliac joint fusion,” “sacroiliac joint arthrodesis,” or “sacroiliac fixation” and “low back pain,” “back pain,” “LBP.”

### 2.1 Inclusion and exclusion criteria

The inclusion criteria will be defined before searching, and the study inclusion eligibility was determined by the following population, intervention, comparator, outcomes, and study design criteria: studies evaluating efficacy and safety between sacroiliac joint fusion and conservative treatment were considered. The age of the patients and follow-up periods were not restricted, and the publication language was limited in English. Studies with less than 10 participants in total were excluded.

### 2.2 Data extraction and quality assessment

Two investigators will independently extract the relevant data from each study, which included the first author's name, year of publication, country, study design, details of the intervention and control including gender, age, number of participants, etc, and the follow-up duration and outcome measurements for each study. Any uncertainty will be discussed by 2 reviewers and resolved by consensus with discussion with another reviewer. We will contact the corresponding authors of the included RCTs to obtain any missing data when necessary. The Cochrane Collaboration tool will be used to assess the methodological quality and risk of bias of the included studies, including randomization, allocation concealment, blinding method, selective reporting, group similarity at baseline, incomplete outcome data, compliance, timing of outcome assessments, and intention-to-treat analysis. The Grading of Recommendations Assessment, Development and Evaluation approach will be used to evaluate the quality of evidence of the included studies. Reviewers will take into account limitations of the study, inconsistencies, indirect evidence, inaccuracies, and publication bias.

### 2.3 Outcome measures

The primary outcome measures that will be evaluated in our review included visual analogue scales (VAS) to assess pain intensity of low back. Oswestry Disability Index (ODI) to evaluate the function and complications during treatment to evaluate the safety. The secondary outcomes will be Shot Form-36, recurrence rate of LBP, and satisfaction of participants.

### 2.4 Statistical analysis and data synthesis

The meta-analyses will be performed using Review Manager (Revman Version 5.3., the Cochrane Collaboration, Oxford, UK). Given the characteristics of the data extracted for the review, continuous outcomes will be expressed as the mean difference (MD) with 95% confidence intervals (CIs). An assumption that the standard deviations (SDs) of outcome measurements are the same in both groups will be required in all cases, and the standard deviation would then be used for both intervention groups. Heterogeneity will be assessed using the *I*^2^ statistic. *I*^2^ ≥ 50% represented high heterogeneity. To detect the impact of each data set on the overall effects of the analyses, sensitivity analysis will be performed by sequentially deleting a single study involved in the meta-analysis. Subgroup analysis will be performed based on the different follow-up periods. Risk ratios (RRs) with a 95% CI were used to assess dichotomous outcomes. The inverse variance and Mantel–Haenszel methods will be used to combine separate statistics. We will evaluate whether asymmetry was due to publication bias or to a relationship between the trial size and effect size using funnel plots. A *P* value < .05 will be considered statistically significant.

### 2.5 Patient and public involvement

No patients will be involved in this study.

## 3 Discussion

The primary objective of this study was to determine whether sacroiliac joint fusion was superior than conservative treatment for patients with LBP in pain and function. The result of this study will illustrate whether there is significant difference between 2 treatments. Whereas acceptance of the SI joint as a potential source of low back pain has grown, confirming that it is the cause of a patient's pain remains a diagnostic challenge.^[18]^ There is lacking of gold standard in diagnosis of LBP attributed to the sacroiliac joint. Moreover, some patients in the diagnosis of the disease and performed fusion surgery suffering consistent pain postoperatively and surgical complications cannot to be neglected.^[19]^ Although minimally invasive surgery has become a trend, there are still many problems, the high patient dissatisfaction rate and high incidence of complications indicated that the treatment of LBP attributed to the sacroiliac joint still needs more large sample with high-quality and well design studies to evaluate the effectiveness and safety. Patients with history of lumbar surgery, misdiagnosed, and personal factors may contribute to the effect of different treatment.

Although pain is generally regarded as originating in the lumbar spine, it has been estimated that in 15% to 30% of patients LBP originates from the sacroiliac joint. Conservative therapies for SIJ pain include medication, physiotherapy, and external orthotics such as pelvic belts. Intra-articular steroid injection has been shown to give short-term pain relief and additionally, pain relief confirms the SIJ as the pain generator. Common non-surgical treatments for LBP attributed to the sacroiliac joint include medication management, sacroiliac joint belts, physical therapy, manipulation, intra-articular steroid injections, etc. Periarticular steroids injection was suggested by some clinical trials as obvious short-term effectiveness was found^[20,21]^ and radiofrequency ablation of sacral nerve roots also suggested a positive effect. However, return of pain 6 to 12 months following ablation is common.

Failure of pain relief using conservative therapies may require surgical treatment. A variety of techniques and approaches have been described both open and minimal incision surgery. Sacroiliac joint fusion was first introduced in 1908. However, it was not routinely used because of collateral damage to the surrounding anatomic structures. Therefore, minimally invasive techniques with novel implants including hollow modular anchor screws^[22]^ and triangular titanium implants have been developed that are designed to confer the benefits of permanent SIJ stabilization recently.^[19,23]^

A recently published trial has reported the improvements in pain, disability, and quality of life observed following surgery were significant given the long duration of pain and the high rate of failure to respond to prior non-surgical management. And the study showed marked improvement in all measures in the surgical group with only minor changes in the non-surgical group. And from a safety perspective, the incidence of postoperative outcomes in the surgery group was low, with a low rate of revision surgery.^[10,15,24]^ As for safety, it was reported that complications occurring in both the surgical and non-surgical groups were not remarkably different across treatment groups at month 6.^[10]^ In general, the overall complication rate from surgery was modest and typical of what would be expected from such a minimally invasive procedure. Complications that required surgical revision occurred in 3 subjects (3%) assigned to SIJF and 1 additional subject who underwent fusion surgery as a crossover procedure.^[19]^

Previous studies have demonstrated that direct medical expenses associated with non-surgical treatment were not inconsequential.^[25,26]^ The indirect costs arising from patients who cannot work because of chronic SIJ pain are even higher; to this end, analysis of data from this study suggests that SIJF may improve worker productivity in this population,^[27]^ indicating that minimally invasive SIJF may also be cost-effective from a societal perspective.

The effectiveness of non-surgical treatments and sacroiliac joint fusion for LBP attributed to sacroiliac joint remains debatable. Although previous systematic review has assessed the value of SIJF for running injuries, and concluded surgical intervention for LBP attributed to sacroiliac joint is beneficial in a subset of patients.^[13]^ However, varied conservative treatments and surgical methods were measured in previous studies. Moreover, there are lack of well design and long-term follow-up studies. A recently published prospective multicenter randomized controlled trial indicated surgical treatment was associated with large improvements in pain and disability, along with marked decreases in opioid use.^[15]^ It is necessary to further evaluate the effect of non-surgical treatments and sacroiliac joint fusion for patients with LBP attributed to sacroiliac joint pain. We aim to use enough studies to ensure adequate power for the meta-analysis. We expect to systematically assess whether surgical treatment can better release pain and improve functional outcome than non-surgical treatments. This study will include the largest amount of studies that systematically assess the efficacy and safety comparing non-surgical and non-surgical treatment for patients suffered from LBP attributed to sacroiliac joint pain. The results of this review may help to give available suggestions for clinical options.

## Acknowledgments

We thank American Journal Experts for its linguistic assistance during the preparation of this manuscript.

## Author contributions

**Conceptualization:** Yingfeng Cai.

**Data curation:** Haodong Liang.

**Investigation:** Qining Qin, Tianzhao Tian, Baoxin Liu.

**Methodology:** Qining Qin, Baoxin Liu.

**Resources:** Min Shi.

**Software:** Haodong Liang, Min Shi.

**Writing – original draft:** Liye Chen.

**Writing – review & editing:** Yingfeng Cai.
